# Preparation of Mesoporous Silica by Nonionic Surfactant Micelle–Templated Gelation of Na_2_SiO_3_ and H_2_SiF_6_ and Application as a Catalyst Carrier for the Partial Oxidation of CH_4_

**DOI:** 10.1038/s41598-019-50053-y

**Published:** 2019-09-16

**Authors:** Kyeong-Won Park, Jin-Young Kim, Ho-Joon Seo, Oh-Yun Kwon

**Affiliations:** 1 Department of Chemistry and Faculty of General Education, Gyeongsang National University, Jinju, 52828 Republic of Korea; 2 Department of Chemical and Biomolecular Engineering, Chonnam National University, Yosu, Chonnam 59626 Republic of Korea

**Keywords:** Porous materials, Synthesis and processing

## Abstract

Mesoporous silica (MSPN12) was prepared by nonionic surfactant micelle–templated gelation of sodium silicate (Na_2_SiO_3_) and fluorosilicic acid (H_2_SiF_6_) in aqueous solution, characterized by a range of instrumental techniques, and tested as a support for Ni and Rh catalysts in the partial oxidation of methane (POM). Calcined and sintered MSPN12 exhibited well-defined *d*_00l_-spacings (3.5–4.39 nm), narrow pore distributions (2.4–3.1 nm), and large specific surface areas (552–1,246 m^2^ g^−1^), and was found to be highly thermally stable. Microscopic imaging revealed that MSPN12 comprised spherical particles with a uniform diameter of ~0.7 µm, with each particle featuring firm and regular honeycomb-type pores. MSPN12-loaded Ni and Rh maintained stable POM activity at 700 °C during almost 100 h on stream, which were comparable to those for the commercial Rh(5)/Al_2_O_3_ catalyst in terms of methane conversion and H_2_ formation selectivity. Thus, the combination of structural stability and favorable physicochemical properties resulted in good POM performance.

## Introduction

Micro- and mesoporous materials are often used as catalyst supports, adsorbents, and ion exchangers. For example, zeolites are widely applied as adsorbents and catalyst supports because of their acidic properties and ion-exchange ability^1^. However, the use of zeolites as catalysts for heavy oil cracking is hindered by their small pore size (<1 nm). To overcome this problem, researchers at Mobil Oil Corporation^2^ developed MCM-41 and MCM-48. Since then, numerous MCM-41–related materials have been synthesized and applied in the field of nanochemistry as catalysts, adsorbents, and catalyst carriers^3–8^. Typically, these materials are prepared using surfactant micelle–based templating, and their pore properties can therefore be altered by changing micelle structure and morphology, e.g., via variation of surfactant type, molecular structure, concentration, and additives^7,9–11^.

MCM-41–type materials synthesized by cationic surfactant micelle–based templating exhibit a very regular and uniform pore structure and are therefore expected to be superior catalyst carriers. However, low pore wall thickness results in the easy collapse of these pore structures under hydrothermal conditions or at temperatures above 700 °C, which can be overcome through the use of nonionic surfactant micelle–based templates. Nonionic surfactants commonly contain ethylene oxide chains that can participate in dipole interactions with metal hydroxide ions^12–14^. When these ions are stabilized by dipole interactions and solidified via condensation, the pore walls of the resulting mesoporous materials are expected to be thicker than the ethylene oxide chain length.

When such micelle templates are calcined, mesopores form in the alkyl chain–containing core portion, while micropores are formed in ethylene oxide chain–containing pore walls. In this case, microporous pore walls are expected to be very thick, and the corresponding supported catalysts are expected to have large specific surface areas and high thermal stabilities. However, despite these advantages, the synthesis of catalysts via nonionic surfactant–based templating and their applications have been underexplored^5,7,14,15^.

Generally, high-purity mesoporous silica (MS) is prepared from silica gel or tetraethylorthosilicate. However, recent studies utilized sodium silicate (Na_2_SiO_3_)^13,16^ or fluorosilicic acid (H_2_SiF_6_)^15–17^ as precursors to minimize problems such as waste solution treatment, which is time-consuming, expensive, and requires the use of non-aqueous solutions.

Herein, MS synthesized by simultaneous gelation of Na_2_SiO_3_ and H_2_SiF_6_ was tested as a catalyst support for the partial oxidation of methane (POM). In this synthesis, H_2_SiF_6_ acted as an acid and silica source, additionally supplying fluoride anions to promote mineralization. As H_2_SiF_6_ can easily dissolve metal oxides and organometallic compounds, it allows one to introduce various metals in the pore walls of silica skeletons. Additionally, H_2_SiF_6_ is a very cheap raw material, since it is a by-product of phosphate fertilizer production. The reaction between H_2_SiF_6_ and Na_2_SiO_3_ was completed within several seconds, which allowed the synthesis of MS to be carried out as a continuous process. MS synthesized in this manner featured large surface area and high thermal stability, and was concluded to be a superior catalyst support for high-temperature reactions.

In recent studies, hydrogen and CO_2_ are highlighted from economic and environmental aspects. Fuel cells are attractive power supplies for versatile applications of hydrogen. In general, hydrogen is obtained by the steam reforming of methane^18^; recently, renewable oxygenated hydrocarbons such as methanol, ethanol, dimethyl ether (DME), and glycerol have also been used^19,20^. CO_2_ methanation^21,22^ and oxidative dehydrogenation of ethane using CO_2_^23^ are promising routes for converting CO_2_ into fuels and chemicals toward CO_2_ emission control.

In this study, we tested MS as the catalyst carrier for the partial oxidation of methane (POM).

## Materials and Methods

### Materials

Na_2_SiO_3_ (35–38 wt% SiO_2_, Kanto Chemical Co., Japan), H_2_SiF_6_ (35 wt%, Alfa Aesar, England), and polyoxyethylene(12) nonylphenol ether (PN12; Aldrich, USA) were used for the preparation of mesoporous silica. Ni(NO_3_)_2_∙6H_2_O (Aldrich, USA), RhCl_3_∙*x*H_2_O (Aldrich, USA), commercial Rh(5)/Al_2_O_3_ (Aldrich, metal loading = 5 wt%), and ethanol (Aldrich, USA) were used for catalyst preparation.

### Preparation of mesoporous silica (MSPN12)

Aqueous solutions of Na_2_SiO_3_ (10 wt%), PN12 (3.0 wt%), and H_2_SiF_6_ (6.0 wt%) were mixed to achieve a Na_2_SiO_3_: H_2_SiF_6_: H_2_O ratio (w/w) of 1: 20: 3.75: 459. First, the surfactant solution was added to the Na_2_SiO_3_ solution, and the resulting dispersion was homogeneously mixed and stirred at 400 rpm for 0.5 h in a constant-temperature reactor at 50 °C. At this point, pH was measured as 11. Subsequently, the H_2_SiF_6_ solution was added in one portion to the mixed solution maintained at 50 °C, which resulted in the formation of a white precipitate within 5 s and a decrease of pH to 5.0. The obtained precipitate was filtered, dried at 60 °C, and calcined in air at 500 °C for 3 h to remove the surfactant template and afford the MSPN12 molecular sieve with a constant pore size. The thermal stability of MSPN12 was tested by additional 3-h sintering at 700, 800, and 900 °C.

### Preparation of catalysts

Ni(5)/MSPN12 and Rh(5)/MSPN12 catalysts (metal loading = 5 wt% each) were prepared as follows. MSPN12 (1.0 g) was dispersed in 5 mL of ethanolic nickel nitrate (containing 0.06 g of Ni(NO_3_)_2_∙6H_2_O)) or rhodium nitrate (containing 0.01 g of RhCl_3_∙*x*H_2_O) solutions upon 5-h stirring, and the resulting dispersions were evaporated, dried for 24 h at 100 °C, and annealed for 5 h at 500 °C in an electric furnace (Eyela TMF-1000) in air. Ethanol was found to be superior to water, allowing one to realize a more uniform dispersion of metals at the gallery surface. Commercial Rh(5)/Al_2_O_3_ was tested together with the above catalysts for comparison.

### Catalytic reaction

The catalytic reaction was carried out under atmospheric pressure in a fixed-bed flow reactor comprising a quartz tube (10 mm inner diameter) and catalyst powder (0.05 g) held on quartz wool. The CH_4_/O_2_ molar ratio, temperature, pressure, and the gas hourly space velocity (GHSV) of the reactant gas equaled 2, 700 °C, 1 atm, and 7.32 × 10^4^ mL g_cat_^−1^ h^−1^, respectively. The reactor was maintained at the desired temperature with an accuracy of ±1 °C using a proportional-integral-derivative controller and a K-type thermocouple located on the catalyst. The reactants were purged using a pressure regulator attached to the corresponding gas cylinders, and the composition of the reactant mixture was controlled using mass flow meters. The effluent was analyzed using an online gas chromatograph (Shimadzu Co., Model 14B, Japan) equipped with a thermal conductivity detector and Porapak Q and 5 A molecular sieve columns arranged in parallel. The fresh catalyst was reduced in a flow of hydrogen (20 mL min^−1^) at 500 °C for 5 h, and the temperature was subsequently increased to 700 °C at a rate of 10 °C min^−1^ before exposure to reactant gases.

### Catalyst characterization

X-ray diffraction (XRD) patterns were recorded on a Bruker D8 Advance diffractometer (Cu *K*_α_ radiation, *λ* = 1.5406 Å; 40 kV, 40 mA). Scanning electron microscopy (SEM) imaging was carried out using a JEOL JSM-840A microscope. Samples intended for SEM imaging were stuck onto adhesive tape, sputter-coated with gold, and imaged. The transmission electron micrographs were obtained with a JEOL JEM-200 CX microscope operated at 200 kV, using the thin-section technique. The powder samples were embedded in epoxy resin and sectioned with an ultra-microtome. N_2_ adsorption/desorption isotherms were recorded by a Micromeritics ASAP 2020 instrument at −196 °C for samples outgassed in vacuum for 4 h at 300 °C. Specific surface areas were determined using the BET method^24^, and pore size distributions were determined using N_2_ adsorption/desorption data and the BJH method^25^.

## Results and Discussion

**Figure 1:**
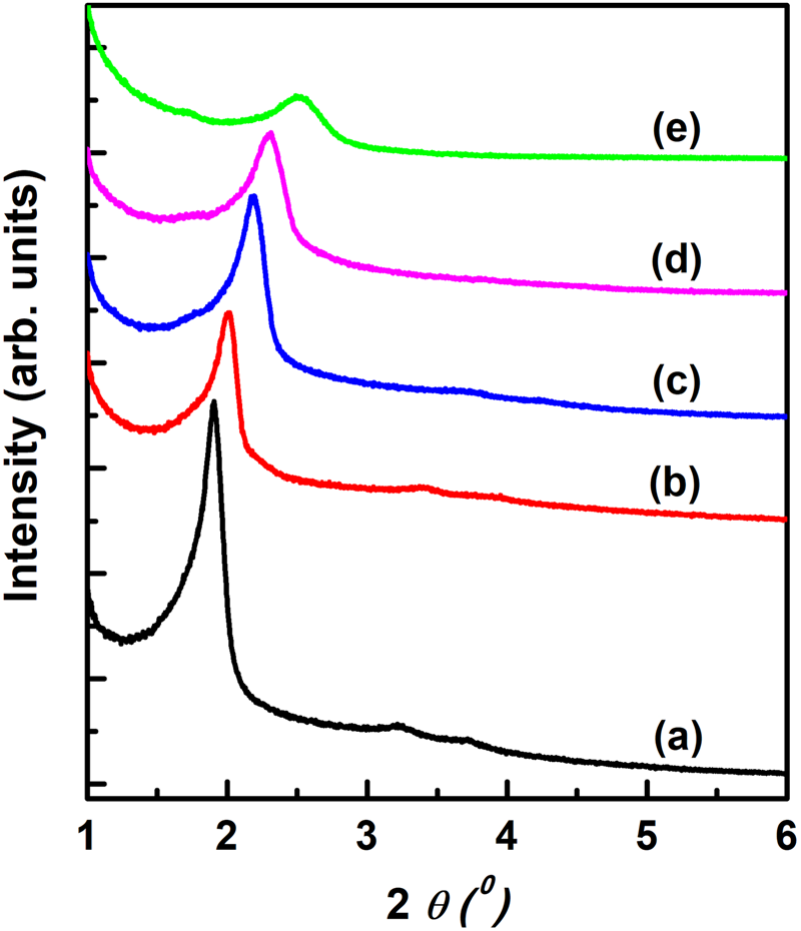
XRD patterns of (**a**) as-prepared MSPN12; (**b**) MSPN12 calcined at 500 °C; MSPN12 calcined under the conditions similar to (**b**) and additionally sintered at (**c**) 700, (**d**) 800, and (**e**) 900 °C.

The gelation of Na_2_SiO_3_ with H_2_SiF_6_ in the presence of a surfactant micelle template was completed within several seconds, affording uniform spherical particles. Figure 1 shows the XRD patterns of MSPN12 sintered for 3 h at 700, 800, and 900 °C after 3-h calcination at 500 °C. MSPN12 calcined at 500 °C showed a well-developed peak corresponding to a *d*-spacing of 4.39 nm, which indicated that surfactant micelles served as templates to form homogeneous pore. The intensity and half-height width of this peak decreased with increasing sintering temperature, reflecting the unclear distinction between pores and pore walls due to the concomitant partial collapse of the pore structure. However, the peak was maintained at 900 °C, which indicated the high thermal stability of MSPN12. The employed nonionic surfactant, PN12, comprised a hydrophilic polyoxyethylene chain [(CH_2_CH_2_O)_12_], which was stabilized by dipole interactions with monomers such as Si(OH)_4_, SiO(OH)_3_^−^, and SiO_2_(OH)_2_^2−^ and solidified via the polycondensation reaction. Consequently, solidification of the monomers between the polyoxyethylene chains results in thicker pore walls than the polyoxyethylene chain length, thus ensuring high thermal stability.

In Na_2_SiO_3_ solutions, Si(OH)_4_ is the predominant species at pH ≤ 7, while in solutions with pH > 9, anionic species such as SiO(OH)_3_^−^ and SiO_2_(OH)_2_^2−^ are converted into grains through hydrolytic condensation reactions^26^. However, in aqueous nonionic surfactant solutions of 3 ≤ pH ≤ 9, Si(OH)_4_ precipitates into SiO_2_ within several seconds. Here, Si(OH)_4_ groups form hydrogen bonds with the oxygens of the polyoxyethylene chains, which promotes the formation of SiO(OH)_3_^−^ anions to induce SiO_2_ precipitation. SiF_6_^2−^ ions in aqueous cationic or nonionic surfactant solutions also precipitate into SiO_2_ within several seconds (even in acidic solutions with pH 3–4)^15,17,27^ which is ascribed to the mineralization-promoting action of F^–^ ions, similar to that of OH^−^ ions. Therefore, the gelation of Na_2_SiO_3_ with H_2_SiF_6_ in the aqueous surfactant solution was promoted by the mineralizing effect of the F^−^ ions and the electrostatic or dipolar interactions between the surfactant micelles and silica monomers (Si(OH)_4_, SiO(OH)_2_^2−^, SiO_2_(OH)_3_^−^, etc.). Silva and Pastore reported that a more uniform pore structure of mesoporous silica is formed in the presence of fluoride ions^28^ and ascribed this behavior to the promotional effect of these ions on the solidification of MSPN12 pore walls, additionally demonstrating that this effect results in enhanced thermal stability.

**Figure 2:**
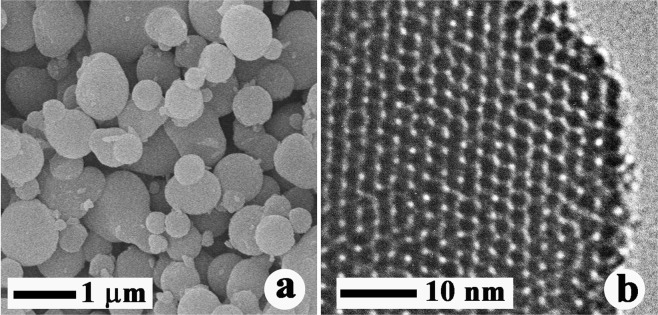
(**a**) SEM and (**b**) TEM images of MSPN12.

Figure 2 shows typical SEM and TEM images of MSPN12, revealing that this material comprised well-separated spherical particles with a uniform diameter of ~0.7 μm, which was ascribed to the fast rate of their formation. Generally, large particles grow around seeds when the rate of crystal seed formation is slow, whereas at higher seed formation rates, the number of these seeds increases so much that the growth of particles stops, which affords smaller particles with a uniform size. In contrast to mesoporous silica obtained using nonionic surfactants, which was reported to exhibit irregular worm hole–type pores opening in all directions^12^, MSPN12 featured pores with a firm and regular honeycomb-like structure.

**Figure 3:**
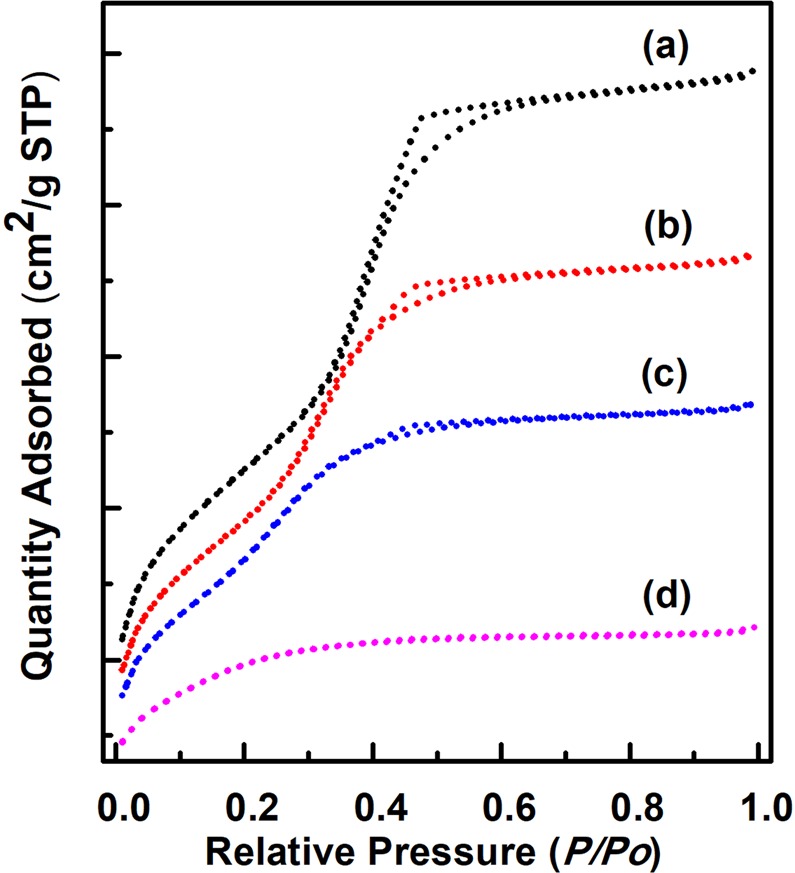
N_2_ adsorption isotherms of calcined MSPN12 sintered at (**a**) 500, (**b**) 700, (**c**) 800, and (**d**) 900 °C.

Figure 3 shows the N_2_ adsorption isotherms of MSPN12 sintered at various temperatures, demonstrating a typical steep increase with mesopore filling at a relative pressure of 0.3–0.4 for all samples.

**Figure 4:**
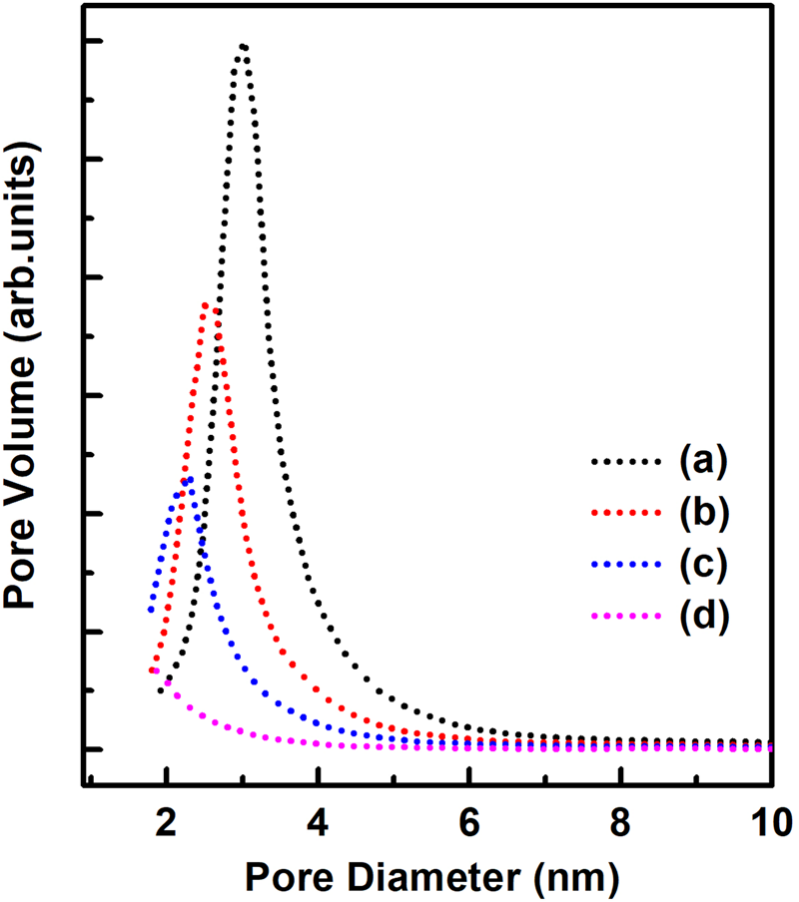
BJH pore size distributions of calcined MSPN12 sintered at (**a**) 500, (**b**) 700, (**c**) 800, and (**d**) 900 °C.

Table 1 lists the specific surface areas obtained using the BET equation, showing that the largest area of 1,246 m^2^ g^−1^ was observed for MSPN12 calcined at 500 °C. This finding was attributed to an increase of micropore surface area due to the effect of polyethylene oxide chains. Upon calcination of micelle templates, mesopores formed in the alkyl chain–containing core portion, while micropores formed in polyethylene oxide chain–containing pore walls, and the resulting mesoporous materials consequently exhibited a considerably large specific surface area. The increase of sintering temperature from 500 to 900 °C brought about a large decrease of surface area from 1,246 to 552 m^2^ g^−1^ due to the thermally induced collapse of micropores. This trend was well reflected in the pore size distributions of MSPN12 (Fig. 4**)**.

**Table 1:** Physical properties of MSPN12 calcined and sintered at various temperatures.

Temperature (°C)	*d*_100_ (nm)	BET surface area (m^2^ g^−1^)	BJH pore diameter (nm)
			adsorption
500	4.39	1,246	3.1
700	4.05	1,088	2.7
800	3.82	882	2.5
900	3.50	552	2.4

**Figure 5:**
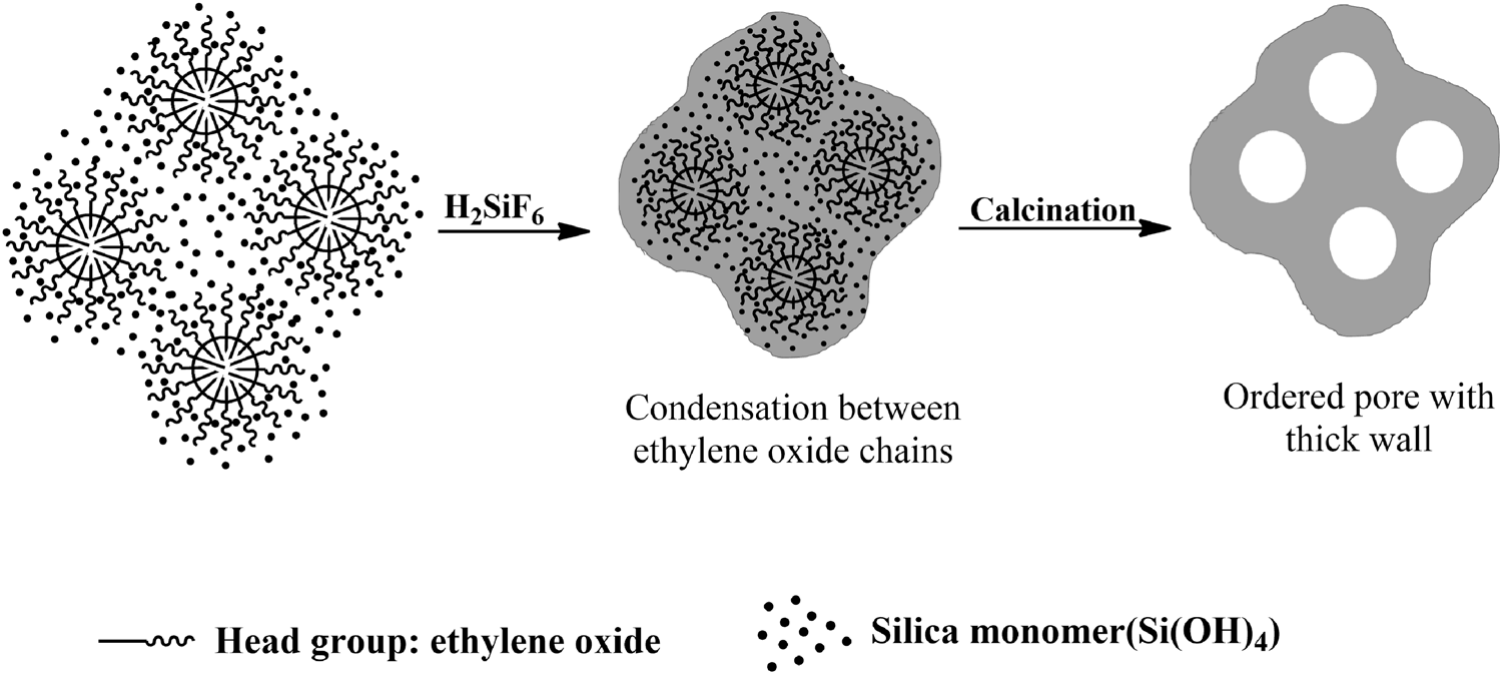
Schematics of mesoporous silica formation via cationic and nonionic surfactant templating.

Figure 5 schematically illustrates the synthesis of MS by nonionic surfactant micelle–based templating, demonstrating that the thick and solid nature of pore walls was caused by the presence of polyethylene oxide chains. Monomers produced in the reaction of Na_2_SiO_3_ and H_2_SiF_6_ (e.g., Si(OH)_4_, SiO_2_(OH)_3_^−^, and SiO(OH)_2_^2−^) were stabilized by dipolar interactions between polyethylene oxide chains at the micelle surface. During the subsequent condensation reaction, this stabilization led to the formation of a thick SiO_2_ skeleton in large spaces between micelles, which endowed MSPN12 with a large surface area and high thermal stability.

**Figure 6:**
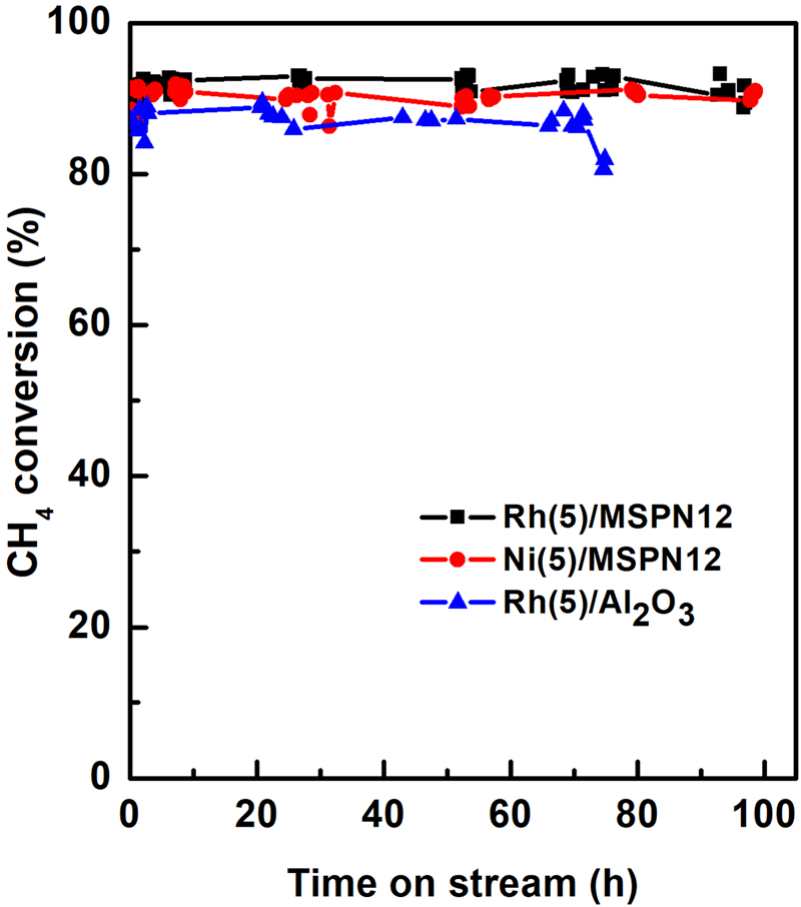
CH_4_ conversions obtained for partial oxidation of methane over Rh(5)/MSPN12, Ni(5)/MSPN12, and Rh(5)/Al_2_O_3_ in a packed bed reactor at *P* = 1 atm, T = 700 °C, CH_4_/O_2_ = 2 (mol/mol), and GHSV = 7.32 × 10^4^ mL g^−1^ h^−1^.

Finally, MSPN12 was examined as a catalyst carrier for POM, with CH_4_ conversions achieved for Ni(5)/MSPN12, Rh(5)/MSPN12, and Rh(5)/Al_2_O_3_ shown in Fig. 6.

**Figure 7:**
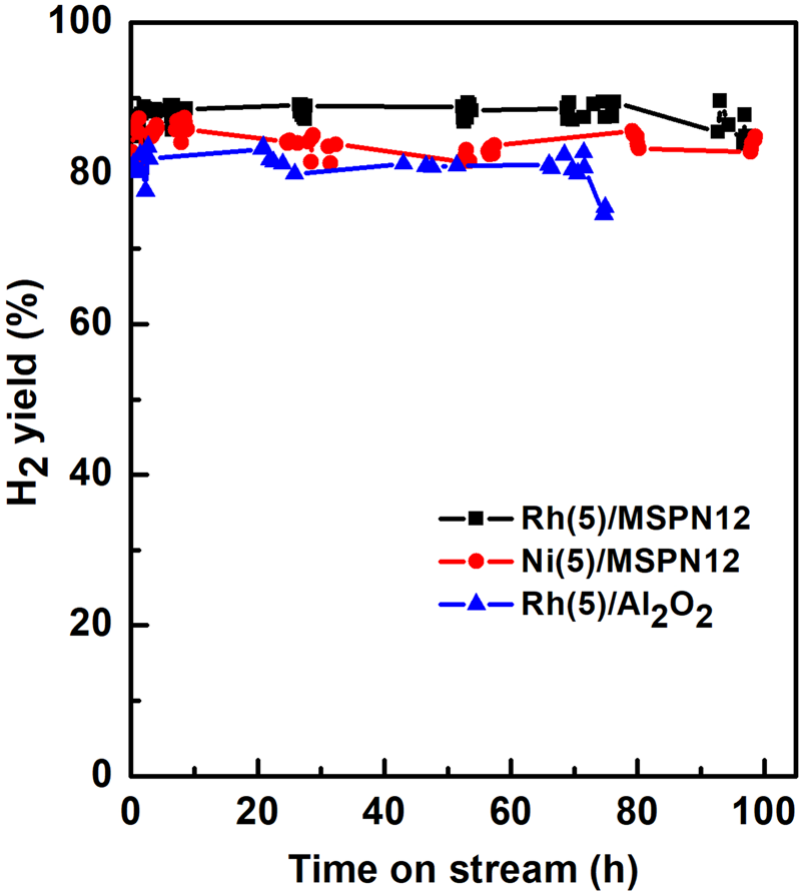
Selectivity of H_2_ formation in partial oxidation of methane over Rh(5)/MSPN12, Ni(5)/MSPN12, and Rh(5)/Al_2_O_3_ in a packed bed reactor at *P* = 1 atm, T = 700 °C, CH_4_/O_2_ = 2 (mol/mol), and GHSV = 7.32 × 10^4^ mL g^−1^ h^−1^.

Ni(5)/MSPN12 and Rh(5)/MSPN12 remained stable for almost 100 h on stream, and CH_4_ conversion (>90%) over these catalysts exceeded that (>80%) over the commercial catalyst. The trends of H_2_ yield, shown in Fig. 7, were similar to those observed for methane conversion, and the high activity and stability of Ni(5)/MSPN12 were consequently concluded to hold great promise for practical applications.

H_2_ yields of nearly 90% were observed for Ni(5)/MSPN12 and Rh(5)/MSPN12, whereas the corresponding yields of (CH)_*x*_ were low, reflecting minimal carbon deposition. Generally, carbon deposition on the catalyst surface results in a decrease of catalytic activity. However, carbon deposition did not disturb the stable performance of MSPN12 catalysts.

**Figure 8:**
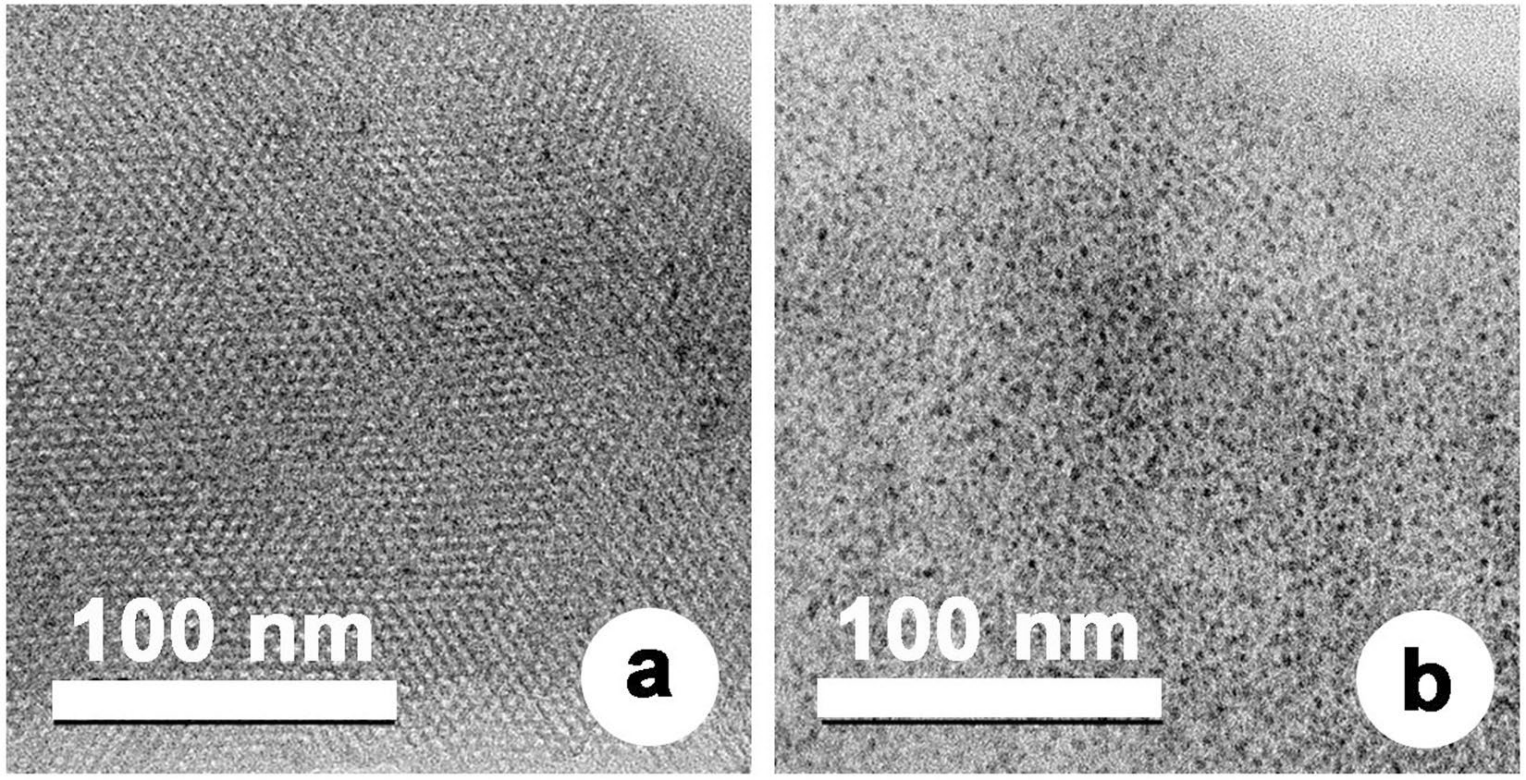
TEM images of (**a**) Ni(5)/MSPN12, and Rh(5)/MSPN12.

In particular, metal particles (Fig. 8) were uniformly dispersed within the mesopores owing to the confinement effect^29^. This uniform dispersion of the metal in the mesopores also contributes to the prolonged maintenance of their catalytic activity. Thus, the obtained results indicated that the large surface area, firm and ordered pore structure, and high thermal stability of MSPN12 contributed to the good POM performance of MSPN12-supported catalysts.

## Conclusion

Herein, mesoporous silica was synthesized through the gelation of Na_2_SiO_3_ and H_2_SiF_6_ in water templated by nonionic surfactant micelles. The above reaction was completed within 5 s and afforded a precipitate comprising well-separated particles with a uniform diameter of ~0.7 µm and a regular honeycomb-like pore structure. Structural analysis of this precipitate allowed it to be classified as a mesoporous molecular sieve. The corresponding specific surface areas were in the range of 552–1,246 m^2^ g^−1^ and depended on sintering temperature, while pore size distributions featured maxima at 2.4–3.1 nm. The synthesized mesoporous silica was shown to be thermally stable up to 900 °C.

Ni- and Rh-impregnated mesoporous silica catalysts remained stable for almost 100 h on stream under the conditions of partial methane oxidation and achieved methane conversions (>90%) exceeding those (>80%) obtained over a commercial Rh(5)/Al_2_O_3_ catalyst, with a similar trend observed for hydrogen yield. The unique properties of mesoporous silica such as its large surface area, ordered pore structure, and high thermal stability were concluded to contribute to the good performance of mesoporous silica–supported catalysts for partial methane oxidation and hold great promise for practical applications.
